# Weight Fallacies

**Published:** 1916-06

**Authors:** 


### Weight Fallacies.

In view of the practical importance of the subject, allusion may again be made to the discounts on weight as an index of nutrition. Ordinary indoor clothing and the things carried in the pockets, weigh 10 pounds or more for a man. The omission or difference in thickness of a single garment is appreciable. The loss over-night by insensible perspiration, urine and respiratory waste and moisture is about 2 pounds. A single evacuation of urine averages half a pound. In the process of "clearing out," the urine, faeces and sweat may account for an apparent loss of 5 pounds or more in a day. On the other hand, a single hearty meal with beverages, may add a pound of solids and 2-4 pounds of liquids in an hour. From the therapeutic standpoint, it should be remembered that gain of weight, even if progressive and considerable is not necessarily advantageous. Every discarded treatment of tuberculosis has had its initial report of gain of weight. As a general rule, the minimum weight in relation to stature and build is the optimum. In other words, leanness nearly to the point of emaciation, is better than moderate obesity.

